# Effects of COVID-19 Disease on DNA Damage, Oxidative Stress and Immune Responses

**DOI:** 10.3390/toxics11040386

**Published:** 2023-04-18

**Authors:** M. Mert Basaran, Merve Hazar, Mehtap Aydın, Gülsüm Uzuğ, İlkima Özdoğan, Emin Pala, Sevtap Aydın Dilsiz, Nursen Basaran

**Affiliations:** 1 Department of Otolaryngology, Faculty of Medicine, Kafkas University, 36000 Kars, Türkiye; 2 Department of Pharmaceutical Toxicology, Faculty of Pharmacy, İbrahim Cecen University, 04100 Ağrı, Türkiye; mrvhzr56@gmail.com; 3 Department of Pharmaceutical Toxicology, Faculty of Pharmacy, Hacettepe University, 06100 Ankara, Türkiye; sevtapay@hacettepe.edu.tr; 4 Department of Infectious Diseases and Clinical Microbiology, Health Sciences University, Ümraniye Training and Research Hospital, 34764 İstanbul, Türkiye; mehtapaydin10@gmail.com (M.A.); gulsumcam25@gmail.com (G.U.); ilkima.ozdogan95@gmail.com (İ.Ö.); 5 Department of Family Medicine, Health Sciences University, Ümraniye Training and Research Hospital, 34764 İstanbul, Türkiye; eminpala72@gmail.com; 6 Department of Pharmaceutical Toxicology, Faculty of Pharmacy, Başkent University, 06490 Ankara, Türkiye

**Keywords:** SARS-CoV-2 infection, COVID-19, DNA damage, oxidative stress

## Abstract

Coronavirus disease 2019 (COVID-19) has posed a great threat to public health and has caused concern due to its fatal consequences over the last few years. Most people with COVID-19 show mild-to-moderate symptoms and recover without the need for special treatment, while others become seriously ill and need medical attention. Additionally, some serious outcomes, such as heart attacks and even stroke, have been later reported in patients who had recovered. There are limited studies on how SARS-CoV-2 infection affects some molecular pathways, including oxidative stress and DNA damage. In this study, we aimed to evaluate DNA damage, using the alkaline comet assay, and its relationship with oxidative stress and immune response parameters in COVID-19-positive patients. Our results show that DNA damage, oxidative stress parameters and cytokine levels significantly increased in SARS-CoV-2-positive patients when compared with healthy controls. The effects of SARS-CoV-2 infection on DNA damage, oxidative stress and immune responses may be crucial in the pathophysiology of the disease. It is suggested that the illumination of these pathways will contribute to the development of clinical treatments and to reduce adverse effects in the future.

## 1. Introduction

Coronavirus disease 2019 (COVID-19), which spread from China to many countries, was declared by the World Health Organization (WHO) as a pandemic in 2020 [1]. It has posed a great health threat to the public because of its fatal outcomes. It has been observed that 20–60% of patients with COVID-19 either have mild symptoms or are asymptomatic [2]. However, even more than a year after acute infection, symptoms called long COVID-19 or post-COVID-19 syndrome were reported by the WHO in many patients who manifested mild symptoms [3,4]. Therefore, it is necessary to further study the long-term effects of this severe acute respiratory syndrome coronavirus 2 (SARS-CoV-2)-induced COVID-19 infection. The complications and clinical evidence of COVID-19 are not clearly understood, and the pathogenesis is still not fully elucidated. Studies revealing the pathogenesis of COVID-19 disease show that not all infected patients develop severe respiratory disease. Several biomarkers of cellular immune response, inflammation and oxidative stress have been used as severity markers in COVID-19 patients. Additionally, some studies show that the release of cytokines is excessively increased in COVID-19 infection. It is known that respiratory viral infections can result in genomic instability by causing excessive cytokine release and by increasing the levels of inflammation, redox and immune response instability [5,6,7,8].

On the other hand, the risks of developing some forms of cancer are shown to be increased by some infectious diseases [9]. As a result of the induction of chronic inflammation or genotoxic damage, viral infections have been suggested to be responsible of more than 10% of all human cancers. These virally induced cancers mainly occur in immunodeficient or immunocompromised people [10]. It is known that Epstein–Barr virus (EBV) can cause many cancers, such as Burkitt’s lymphoma, Hodgkin’s lymphoma, naso-pharyngeal carcinoma and gastric cancers [11,12]. Human papillomavirus (HPV) can lead to cervical cancers [13]. Hepatitis C virus is one of the major risk factors for liver cancer [14]. Additionally, chronic inflammation is associated with the production of free reactive oxygen radicals that can promote the development of some types of cancer and vascular lesions [15,16]. It was reported that patients infected with the influenza virus have a high level of oxidized biomolecules, such as DNA, lipids and proteins [17,18]. Reactive oxygen species (ROS) can induce several types of damage to DNA, including DNA-protein crosslinks, single- and double-strand breaks, and base (such as oxidized guanine species) and sugar oxidation products (breaks in the sugar phosphate DNA backbone, such as 8,5′-cyclopurine 2′-deoxynucleosides) [19,20,21]. Although it has not been proven yet and more mechanistic research studies are required, oxidative stress is suggested to play an important role in COVID-19 pathogenesis [22,23,24]. Oxidative stress is defined as the imbalance between the presence of antioxidants and pro-oxidants in a biological system [18,25]. The development of oxidative stress in patients with various viral infections has been shown in some studies, and it was suggested that oxidative stress impacts the disease pathogenesis mostly by impairing immune functions, apoptosis and inflammatory responses [26,27,28]. Patients with pre-existing diseases (such as diabetes; hypertension; and pulmonary, cardiac, and kidney failure) that are suggested to cause an increase in oxidative stress seem to be at higher risk of having serious outcomes of the infection [29,30]. Patients with COVID-19 are also suggested to develop a wide range of neurological symptoms, including ischemic stroke, meningoencephalitis and some forms of encephalopathy [31,32]. Severe COVID-19 infection was suggested to cause a cytokine storm, which is an imbalanced and uncontrolled cytokine response, which also initiates exuberant endothelial inflammatory reactions, and vascular thrombosis. These events may lead to the development of acute respiratory distress syndrome (ARDS), which is the major reason of death in COVID-19 patients [33]. Moreover, since oxidative DNA damage has long been associated with increased risks of developing neurodegenerative diseases and several types of cancer [34,35], the possibility of developing chronic diseases in COVID-19 patients should be addressed in future studies. In this study, we aimed to evaluate DNA damage, using the alkaline comet assay, and its relationship with oxidative stress and immune response parameters in COVID-19-positive patients with mild or severe symptoms and to compare the results with healthy controls. In addition, the differences in the degree of DNA damage and certain biochemical parameters between COVID-19 patients and healthy controls were also evaluated.

## 2. Materials and Methods

### 2.1. Patients and Experimental Design

The study population comprised a total of 50 patients with positive COVID-19 findings (12 patients with severe symptoms and 38 patients with mild symptoms) who came to Health Sciences University Ümraniye Training and Research Hospital with respiratory system or fever complaints with the suspicion of COVID-19 and 50 heathy controls of comparable age, gender, lifestyle and smoking habits living in the same area and without a history of chemical exposure, chronic disease or drug use (Table 1).

All volunteers were given information about the aim of the study, and their written consent was obtained. Before sample collection, a detailed questionnaire about the health status, medical history, and alcohol and smoking habits of patients and controls were obtained. Subjects who reported chronic diseases, radiotherapy or chemotherapy were excluded. No alcohol intake was reported in all the study groups.

This study was approved by the local ethics commission of Health Sciences University Ümraniye Training and Research Hospital (Date: 12 May 2020; No. B.10.1.TKH.4.34.H.GP.0.01) and was conducted in accordance with the ethical standards of the 1964 Declaration of Helsinki.

### 2.2. Preparation of Samples

Peripheral blood samples totaling 10 mL were taken from each volunteer. All blood samples were maintained at +4 °C and processed within two hours. Two-milliliter blood samples collected in tubes without anticoagulant were centrifuged for 10 min at 3000 rpm to separate the serum. The serum samples were used for the measurement of biochemical parameters such as serum C-reactive protein (CRP), ferritin, d-dimer, fibrinogen, aspartate aminotransferase (AST) and alanine aminotransferase (ALT). Two-milliliter blood samples collected in EDTA-containing tubes were centrifuged for 10 min at 3000 rpm to separate the plasma. The plasma samples were used for the analysis of hemograms, including basophils (BASO), eosinophils (EOS), hematocrit (HCT), hemoglobulin (Hg), lymphocytes (LYM), mean corpuscular hemoglobin (MCH), mean corpuscular hemoglobin concentration (MCHC), mean corpuscular volume (MCV), monocytes (MONO), mean platelet volume (MPV), neutrophils (NEU), procalcitonin (PCT), platelet distribution width (PDW), platelets (PLT), red-cell distribution width standard deviation (RDW-SD), white blood cells (WBCs) and red blood cells (RBCs). Six-milliliter blood samples were taken into tubes containing sodium heparin. Plasma was obtained from 5 mL of the sample for the analysis of oxidative stress and immune parameters (malondialdehyde (MDA), glutathione (GSH), glutathione peroxidase (GPx), superoxide dismutase (SOD), catalase (CAT), interleukin (IL)-17, IL-23 and IL-27). The plasma samples were stored at −80 °C until the day of analysis. A volume of 1 mL of peripheral lymphocytes was isolated from the remaining heparinized blood samples and studied within 24 h for the analysis of DNA damage.

### 2.3. Determination of Biochemical Parameters

Hemograms (whole blood), CRP, ferritin, d-dimer, fibrinogen, AST and ALT were measured with the turbidimetric method using a Sysmex XN-2000 (Sysmex Europe GMBH, Norderstedt, Germany) hematology autoanalyzer. The samples were analyzed in duplicate. The biochemical parameters were expressed as 10^3^/µL for WBCs, PLT, PCT, NEU, MO, LYM, EOS and BASO; 10^6^/µL for RBCs; g/dL for Hg and MCHC; femtoliter (fL) for MCV, RDW-SD, PDW and MPV; picogram (pg) for MCH; % for HCT; mg/L for CRP and fibrinogen; ng/mL for ferritin; µg/mL for d-dimer; U/dL for AST and ALT.

### 2.4. Determination of Oxidative Stress and Immune Parameters

Oxidative stress and immune parameters including MDA, GSH, GPx, SOD, CAT, IL-17, IL-23 and IL-27 were spectrophotometrically determined using ELISA kits by Biossay Technology Laboratory (BT LAB) (Shanghai Korain Biotech Co., Ltd., Shanghai, China) following the manufacturer’s directions. Additional reagents were purchased from Sigma-Aldrich (St. Louis, MO, USA). For spectrophotometric and spectrofluorometric measurements, SpectraMaxM2 (Molecular Devices, Sunnyvale, CA, USA) and, for quantification, SoftMax Pro Software 7.1 (Molecular Devices) were used. The samples were studied in duplicate. The parameters were expressed as nmol/L for MDA; ng/mL for GSH and SOD; µU/mL for GPx; KU/L for CAT; ng/L for IL-17, Il-23 and IL-27.

### 2.5. Determination of DNA Damage

The basic alkaline single-cell gel electrophoresis technique (comet assay) by Singh et al. [36] as further specified by Collins et al. [37] was performed to determine DNA damage. Lymphocytes were separated using the Ficoll–Hypaque density gradient technique by centrifugating the heparinized peripheral blood samples [38]. After washing with phosphate-buffered saline (PBS) buffer twice, the cell concentrations were adjusted to about 2 × 10^5^/mL in PBS buffer. A volume of 50 µL of cell suspension (10^4^ cells per slide) was mixed with 150 µL of 1% low-melting-point (LMP) agarose and then embedded on slides pre-coated with 1% normal-melting-point (NMP) agarose. The slides were lysed with freshly prepared ice-cold lysis solution (2.5 M NaCl, 0.1 M EDTA, 0.1 M Tris and 1% sodium sarcosinate; pH 10.0) with 1% Triton X-100 and 10% DMSO for 1 h at 4 °C to form nucleoids containing supercoiled loops of DNA linked to the nuclear matrix. Then, they were removed from the lysis solution, drained and left in the electrophoresis solution (0.1 M sodium EDTA and 0.3 M NaOH; pH 13.0) for 20 min at 4 °C to allow the unwinding of DNA and the expression of alkali-labile damage to be achieved. Electrophoresis was carried out for 20 min at 4 °C with a current of 25 V (300 mA). After electrophoresis, the slides were immediately washed with distilled water to remove residues of detergents and salts. The slides were then neutralized three times for 5 min in Tris buffer (pH 7.5) and incubated in 50%, 75% and 98% alcohol for 5 min. The microscopic slides were stored under dry conditions until analysis.

The slides were stained with ethidium bromide (20 μg/mL in distilled water). Image analyses of the slides were carried out using a green light-fluorescence microscope (LeicaVR M205 FCA; Wetzlar, Germany) connected to a charge-coupled device camera and a personal computer-based analysis system (Comet Assay IV™ software; Instem-Perceptive Instruments Ltd., Suffolk, Halstead, UK). One-hundred cells from each of the duplicate slides were examined by a well-trained comet specialist at 400× magnification in order to visualize DNA damage. The extent of DNA damage was expressed as the percentage of DNA in the tail (tail intensity). Nucleoids containing >80% of DNA in the tail region were not included to represent DNA damage that could have resulted from cytotoxicity [25].

### 2.6. Statistical Analysis

The statistical evaluation of the data set was carried out using IBM SPSS software (IBM^®^ SPSS Statistics 23.0) for Windows. The samples were studied in duplicate. The results were presented as means ± standard deviation (SD) (min–max) and the number of cases per cent (%) for continuous variables and categorical variables, respectively.

Statistical differences between groups with normal distribution were determined using the one-way analysis of variance (ANOVA) test. The post hoc analysis of group differences was performed with the least significant difference (LSD) test. Statistical differences between groups without normal distribution were analyzed using the Mann–Whitney U test for two groups and the Kruskal–Wallis test for more than two groups. The z-test was applied to the statistical analysis of categorical values.

The homogeneity of the variance was tested using the Levene test. The Kolmogorov–Smirnov test was used to determine the normality of the distribution. The magnitude of linear relationships was calculated using Pearson correlation analysis. A *p*-value of less than 0.05 was considered statistically significant.

## 3. Results

### 3.1. Characteristics of the Study Population

The characteristics (age, body mass index, gender, smoking habits, fever, cough, respiratory symptoms and hospitalization) of patients and healthy controls are given in Table 1. In the evaluation of hospitalization, CRP (>50 mg/L), d-dimer (>550 µg/mL), ferritin (>500 ng/mL), fibrinogen (>400 mg/dL) and lymphopenia (lymphocytes < 1 × 10^3^/µL) values were especially considered together with the symptoms.

The mean ages of patients and controls were 43.58 ± 16.17 years (range of 18–73) and 41.16 ± 14.28 years (range of 18–75), respectively. The average cigarette consumption rates were 8.23 ± 7.49 cigarettes/day and 11.72 ± 19.84 cigarettes/day in smoking patients (34%) and healthy smokers (40%), respectively. There were no statistically significant differences between patients and healthy controls in terms of age, body mass index, gender and smoking habits (Table 1).

Of all the patients, 40% had a fever; a total of 52% had a cough; and 24% had respiratory distress symptoms. While 24% of patients were admitted to hospital, 76% of patients with mild symptoms did not require hospitalization (Table 1).

### 3.2. Hemograms and Biochemical Parameters

The hemograms and biochemical parameters of the study groups are presented in Table 2 and Table 3, respectively.

There was no difference between the study groups in terms of the hemograms, including WBC, RBC, Hg, MCV, MCH, MCHC, HCT, RDW-SD, PDW, MPV, PCT, NEU, MO and BASO levels (*p* > 0.05). Compared with healthy controls, lymphocyte and eosinophil levels decreased in all patients; moreover, these levels decreased more dramatically in patients with severe symptoms (*p* < 0.05) (Table 2).

The lymphocyte levels of the patient group were 47.52% (0.52-fold) lower than those of the control group (*p* < 0.05). The lymphocyte levels of patients with severe symptoms and mild symptoms were 69.4% (0.31-fold) and 40.8% (0.59-fold) lower than those of the control group, respectively (*p* < 0.05). The lymphocyte levels of patients with severe symptoms were 48.4% (0.52-fold) lower than those of patients with mild symptoms (*p* < 0.05) (Table 2).

The eosinophil levels of the patient group were 50.0% (0.50-fold) lower than those of the control group (*p* < 0.05). The eosinophil levels of patients with severe symptoms and mild symptoms were 88.9% (0.11-fold) and 38.9% (0.61-fold) lower than those of the control group, respectively (*p* < 0.05). The eosinophil levels of patients with severe symptoms were 81.8% (0.18-fold) lower than those of patients with mild symptoms (*p* < 0.05) (Table 2).

The PLT levels were not different between the patient and control groups, and between the severe- and mild-symptom groups (*p* > 0.05); however, the PLT levels of patients with severe symptoms were 36.5% (1.37-fold) higher than those of the healthy controls (*p* < 0.05) (Table 2).

Compared with healthy controls, the CRP, ferritin, d-dimer and fibrinogen levels were found to be high in all patients with COVID-19; moreover, the CRP, ferritin and d-dimer levels of patients with severe symptoms increased more dramatically (*p* < 0.05) (Table 3). The CRP levels of the patient group were 1261.4% (13.61-fold) higher than those of the control group (*p* < 0.05). The CRP levels of patients with severe symptoms and mild symptoms were 4178.7% (42.79-fold) and 340.1% (4.40-fold) higher than those of the control group, respectively (*p* < 0.05). The CRP levels of patients with severe symptoms were 872.3% (9.72-fold) higher than those of patients with mild symptoms (*p* < 0.05) (Table 3).

The ferritin levels of the patient group were 373.3% (4.73-fold) higher than those of the control group (*p* < 0.05). The ferritin levels of patients with severe symptoms and mild symptoms were 962.5% (10.62-fold) and 187.3% (2.87-fold) higher than those of the control group, respectively (*p* < 0.05). The ferritin levels of patients with severe symptoms were 269.8% (3.70-fold) higher than those of patients with mild symptoms (*p* < 0.05) (Table 3).

The d-dimer levels of the patient group were 148.7% (2.49-fold) higher than those of the control group (*p* < 0.05). The d-dimer levels of patients with severe symptoms and mild symptoms were 482.7% (5.83-fold) and 43.1% (1.43-fold) higher than those of the control group, respectively (*p* < 0.05). The levels of patients with severe symptoms were 307.1% (4.07-fold) higher than those of patients with mild symptoms (*p* < 0.05) (Table 3).

The fibrinogen levels of all patients with COVID-19 and patients with severe symptoms were 41.7% (1.42-fold) and 86.6% (1.87-fold) higher than those of the control group, respectively (*p* < 0.05). The levels were not different between patients with mild symptoms and healthy controls or between the severe- and mild-symptom groups (*p* > 0.05) (Table 3).

The AST levels were not different between patients and controls or between patients with severe and mild symptoms (*p* > 0.05); however, the AST levels significantly increased in patients with severe symptoms (74.8%) (1.75-fold) when compared with healthy controls (*p* < 0.05) (Table 3).

There was no difference in ALT levels between the study groups (*p*>0.05) (Table 3).

### 3.3. Oxidative Stress and Immune Parameters

The oxidative stress parameters and the immune biomarkers of patients and controls are given in Table 4.

The MDA levels of the patient group were 252.2% (3.52-fold) higher than those of the control group (*p* < 0.05). The MDA levels of patients with severe symptoms and mild symptoms were 679.8% (7.80-fold) and 117.4% (2.17-fold) higher than those of the control group, respectively (*p* < 0.05). The MDA levels of patients with severe symptoms were 258.7% (3.59-fold) higher than those of patients with mild symptoms (*p* < 0.05) (Table 4).

The GPx levels of the patient group were 352.7% (4.53-fold) higher than those of the control group (*p* < 0.05). The GPx levels of patients with severe symptoms and mild symptoms were 858.1% (9.58-fold) and 193.0% (2.93-fold) higher than those of the control group, respectively (*p* < 0.05). The GPx levels of patients with severe symptoms were 227.0% (3.27-fold) higher than those of patients with mild symptoms (*p* < 0.05) (Table 4).

The SOD levels of the patient group were 301.5% (4.02-fold) higher than those of the control group (*p* < 0.05). The SOD levels of patients with severe symptoms and mild symptoms were 750.4% (8.50-fold) and 160.0% (2.60-fold) higher than those of the control group, respectively (*p* < 0.05). The SOD levels of patients with severe symptoms were 227.1% (3.27-fold) higher than those of patients with mild symptoms (*p* < 0.05) (Table 4).

The CAT levels of the patient group were 255.1% (3.55-fold) higher than those of the control group (*p* < 0.05). The CAT levels of patients with severe symptoms and mild symptoms were 581.7% (6.82-fold) and 152.5% (2.52-fold) higher than those of the control group, respectively (*p* < 0.05). The CAT levels of patients with severe symptoms were 170.0% (2.70-fold) higher than those of patients with mild symptoms (*p* < 0.05) (Table 4).

The IL-17 levels of the patient group were 253.1% (3.53-fold) higher than those of the control group (*p* < 0.05). The IL-17 levels of patients with severe symptoms and mild symptoms were 669.5% (7.69-fold) and 121.6% (2.22-fold) higher than those of the control group, respectively (*p* < 0.05). The IL-17 levels of patients with severe symptoms were 247.2% (3.47-fold) higher than those of patients with mild symptoms (*p* < 0.05) (Table 4).

The IL-23 levels of the patient group were 281.6% (3.82-fold) higher than those of the control group (*p* < 0.05). The IL-23 levels of patients with severe symptoms and mild symptoms were 747.9% (8.48-fold) and 134.3% (2.34-fold) higher than those of the control group, respectively (*p* < 0.05). The IL-23 levels of patients with severe symptoms were 161.8% (3.62-fold) higher than those of patients with mild symptoms (*p* < 0.05) (Table 4).

The IL-27 levels of the patient group were 288.7% (3.89-fold) higher than those of the control group (*p* < 0.05). The IL-27 levels of patients with severe symptoms and mild symptoms were 731.9% (8.32-fold) and 148.7% (2.49-fold) higher than those of the control group, respectively (*p* < 0.05). The IL-27 levels of patients with severe symptoms were 234.5% (3.34-fold) higher than those of patients with mild symptoms (*p* < 0.05) (Table 4).

### 3.4. The Alkaline Single-Cell Gel Electrophoresis Technique (Comet Assay)

**Figure 1:**
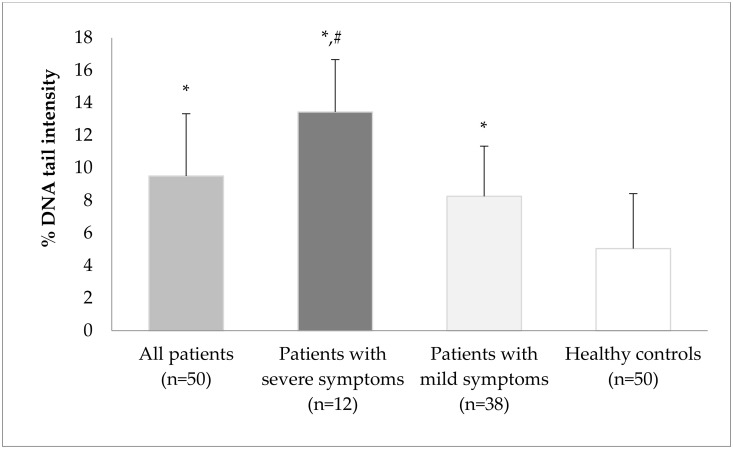
DNA damage in lymphocytes of the study groups using the alkaline comet assay. DNA damage was expressed as DNA tail intensity (% of DNA in the tail) in lymphocytes. The values are given as means ± standard error mean (min–max). * *p* < 0.05, compared with healthy controls; # *p* < 0.05, patients with severe symptoms compared with patients with mild symptoms.

DNA damage expressed as DNA tail intensity (% of DNA in the tail) in lymphocytes is shown in Figure 1. DNA damage increased in all patients with COVID-19; moreover, DNA damage in patients with severe symptoms increased more dramatically than in the control group (*p* < 0.05).

DNA damage in the patient group was 94.9% (1.95-fold) higher than that in the control group (*p* < 0.05). The tail intensity was 175.4% (2.75-fold) and 69.3% (1.69-fold) higher in patients with severe and mild symptoms, respectively, than in the control group (*p* < 0.05). DNA tail intensity in severe patients was 62.7% (1.63-fold) higher than that in patients with mild symptoms (*p* < 0.05). (Figure 1).

There was no correlation between DNA damage and GSH levels, while strong, positive correlations between DNA damage and the levels of MDA (r = 0.803), GPx (r = 0.766), SOD (r = 0.763), CAT (r = 0.793), IL-17 (r = 0.739), IL-23 (r = 0.753) and IL-27 (r = 0.979) were observed in the study groups. Moreover, moderate, positive correlations between DNA damage and CRP levels (r = 0.694), and between DNA damage and fibrinogen levels (r = 0.604) were found in the study groups.

## 4. Discussion

The new corona virus 2019 (COVID-19) infection, which has threatened the health of many people in many countries, is the main pandemic of this century. It affected the whole world and had a wide range of consequences from mild illness to severe infection resulting in death. Oxidative stress, defined as the imbalance between the production of ROS and their elimination by antioxidants, is thought to be associated with the pathogenesis and effects of COVID-19 [22]. It is suggested to play an important role in severe acute respiratory syndrome coronavirus (SARS-CoV) infection [39,40]. There are few studies on how SARS-CoV-2 infection affects the pathways of apoptosis, oxidative stress and genotoxicity. Since coronaviruses can induce oxidative stress and DNA damage and can impair DNA repair mechanisms, there is the possibility of the risk of the future development of chronic diseases in recovered COVID-19 patients. If there is a carcinogenic risk associated with SARS-CoV-2, the implications for public health are enormous, as infected patients would have to be closely monitored during long follow-up periods. Additional research to identify or exclude the possibility of persistent infection is crucial to prevent potential complications of COVID-19 in the future [41,42].

Since there are very few clinical studies assessing the role of immune and oxidative stress-related biomarkers in COVID-19 pathogenesis, in our study, we aimed to investigate the effects of COVID-19 on some important biochemical, immune and oxidative stress biomarkers in COVID-19-positive patients. We evaluated the biochemical parameters of 50 patients, of which 12 patients had severe symptoms and 38 patients had mild symptoms, and compared the results with 50 healthy controls. Our findings of increased levels of CRP, ferritin, d-dimer, fibrinogen, AST and platelets, and lower levels of lymphocytes and eosinophils in patients with severe symptoms are consistent with the study by Martins-Filho et al. [43], who observed increased levels of IL-6, CRP, ferritin and procalcitonin; increased erythrocyte sedimentation rate; decreased CD4 and CD8 cell counts; changes in biochemical indices such as albumin, blood urea nitrogen, creatinine, creative kinase, hypersensitive cardiac troponin I and lactate dehydrogenase; and coagulation abnormalities, including prolonged prothrombin time, increased d-dimer and thrombocytopenia. They suggested that these changes are important predictors of mortality in patients with COVID-19. In our study, apart from increased levels of CRP, ferritin, d-dimer and fibrinogen, we also found higher levels of AST in patients with severe symptoms than in healthy controls. Liao et al. [44] investigated serum hepatic enzyme activities in 147 COVID-19 patients and evaluated their relationship with illness severity. They also observed abnormal liver functions in patients with severe COVID-19. However, contrary to our findings, they observed a decrease in serum SOD levels. In our study, a significant increase in the SOD levels of patients with severe symptoms was observed. Çakırca et al. [8] also measured higher levels of white blood cells, neutrophils, CRP, procalcitonin, ferritin, fibrinogen and urea, and low levels of lymphocytes and albumin in intensive care unit (ICU) COVID-19-positive patients than in non-ICU patients. They also found significantly lower levels of thiol and total antioxidant status (TAS) but higher levels of total oxidant status (TOS) and oxidative stress index (OSI) in the serum of COVID-19 patients in the ICU than in non-ICU patients, suggesting an increase in oxidative stress and a decrease in antioxidant levels in severe COVID-19-infected patients. Our study also revealed a significant increase in MDA levels in COVID-19 patients compared with healthy controls, and the observed increase was more pronounced in patients with severe symptoms, suggesting an increase in lipid peroxidation and excessive oxygen production. However, in our study, although the levels of glutathione (GSH) were not found to be different between COVID-19-positive patients and healthy controls, we observed increased activities of antioxidant enzymes such as superoxide dismutase (SOD), catalase (CAT) and glutathione peroxidase (GPx), and the increases were more significant in patients with severe symptoms. Since the samples from the patients were taken at the time of diagnosis of the disease, it seemed that the antioxidant parameters were increased in COVID-19 patients, suggesting increases in defense mechanisms against lipid peroxidation at the onset of disease in patients. Mehri et al. [24] investigated the oxidative stress markers in 24 COVID-19 patients compared to 24 healthy subjects. Similar to our study, their study showed that serum MDA and TOS levels, and CAT and GPX activities were significantly increased in COVID-19 patients. The biochemical function of GPx is to reduce lipid hydroperoxides to their corresponding alcohols and to reduce free hydrogen peroxide to water. The antioxidant enzymes SOT and CAT also protect the body from oxidative damage. There are conflicting data about the activities of antioxidant levels in COVID-19 patients. In a prospective cohort study involving 120 COVID-19-positive patients and 60 healthy controls, neutrophils were found to be higher in the critical group, while lymphocytes were lower in the severe and critical groups than in the mild group. The CRP, ferritin and d-dimer values of severe and critical cases were higher than those of mild COVID-19 cases. MDA, nitric oxide and copper increased, while SOD activity and total antioxidant capacity (TAC) levels were found to be decreased in severe COVID-19 patients. However, vitamin C, GPx and CAT activity were not different between COVID-19 groups and controls. It was suggested that the oxidative and inflammatory damage induced by SARS-CoV-2 may be the result of an imbalance between the amount of ROS and antioxidants [45]. In another study on 120 patients with COVID-19 infection and 60 healthy volunteers, the relationship between the oxidant/antioxidant system and COVID-19 disease severity was investigated. The TAC levels were significantly lower in patients than in healthy individuals, and differences were also found between patients with mild and severe symptoms; however, SOD and CAT activities were not statistically significant. It is suggested that COVID-19 patients may be sensitive to decreased total antioxidant capacity [46]. On the other hand, it was observed that ROS triggered NF-κB-driven pro-inflammatory cytokines, as well as chemokines, resulting in an uncontrolled cytokine storm that induced acute lung damage and acute respiratory distress syndrome in COVID-19-infected patients. Lung tissue damage due to excessive cytokine release can be associated with the pathogenesis of the disease [47]. We found that pro-inflammatory and inflammatory chemokines, such as IL-17, IL-23 and IL-27, were increased in COVID-19-positive patients compared with their healthy controls. In patients with severe symptoms, the increases were found to be more significant. It is suggested that some cytokines, such as IL-23, can promote the progression of cardiovascular diseases such as atherosclerosis, hypertension, aortic dissection, myocardial infarction and acute cardiac injury [48]. In the brain, IL-23 is found to activate the expression of IL-17, which contributes to the inflammatory response and thus plays a key role in secondary brain injury after spontaneous intracerebral hemorrhage [49]. The possibility of developing chronic cardiovascular and neurodegenerative diseases in COVID-19 patients should be addressed in future studies.

To our knowledge, this is the first study who determined IL-17, IL-23 and IL-27. Patients with severe COVID-19 showed significant increases in cytokines, including IL-2, IL-7, IL-10, GSCF, IP10, MCP-1, MIP1A and TNF-α, with the characteristics of a cytokine storm [50]. Monserat et al. [51] studied 62 circulating soluble factors, including innate and adaptive cytokines and their soluble receptors, chemokines, and growth and wound-healing/repair factors, in 286 severe COVID-19 survived patients and compared the results with fatal outcomes. The following were strongly associated with survival: increases in the circulating levels of the sCD40L cytokine; MDC and RANTES chemokines; G-CSF and GM-CSF growth factors, EGF, PDGFAA and PDGFABBB; and tissue-repair factors. By contrast, the following favored fatal outcomes of the disease: large increases in IL-15, IL-6, IL-18, IL-27 and IL-10; sIL-1RI, sIL1RII and sTNF-RII receptors; MCP3, IL-8, MIG and IP-10 chemokines; M-CSF and sIL-2Ra growth factors; and the wound-healing factor FGF2.

In our study, we also evaluated DNA damage and its relationship with oxidative stress and immune response parameters in COVID-19-positive patients with mild or severe symptoms and compared the results with healthy controls. DNA damage increased in all patients with COVID-19; moreover, DNA damage in patients with severe symptoms increased more dramatically than in patients with mild symptoms. Our data showing increased oxidative stress and higher DNA damage in COVID-19-positive patients are consistent with the studies by Bektemur et al. [31], who found induced oxidative stress, DNA damage and inflammation biomarkers in 150 patients diagnosed with COVID-19. They found increased levels of total oxidant status, myeloperoxidase, IL-1β, IL-6 and TNF-α, and decreased total antioxidant status in patients when compared with healthy volunteers. Mihaljevic et al. [32] also found increased DNA damage as measured using the comet assay in 24 severely COVID-19 patients compared with 15 healthy control subjects. They reported that DNA damage was positively correlated with interleukin 6 (IL-6) concentration and negatively correlated with platelet count in COVID-19 patients. The level of DNA damage significantly corresponded to the inflammatory markers and parameters of hemostasis. Tepebaşı et al. [52] studied the effects of inflammation markers (neutrophil–lymphocyte ratio, serum reactive protein, procalcitonin, etc.), IL-10 and IF γ levels, and oxidative stress on DNA damage in 95 patients hospitalized with COVID-19, who were divided into three groups according to the severity of pneumonia—mild, moderate and severe/critical. Total thiol, native thiol and disulfide levels significantly decreased due to the increase in inflammation markers and cytokine levels in severe patients. Similar to our data, their data indicate that an increase in DNA damage was observed due to the increased oxidative stress. High levels of CRP were reported to be associated with high levels of oxidative damage in the DNA of patients with psoriasis, obesity, pancreatic cancer and cardiovascular diseases. Oxidative DNA damage is associated with increased risks of developing chronic diseases and several types of cancer. This possibility of developing chronic diseases cannot be ignored in COVID-19 patients. Oxidative stress plays an important role in COVID-19 pathogenesis, and all of these clinical observations require mechanistic research follow-up. Severe COVID-19 patients with pro-inflammatory and pro-oxidative states may be at increased risk of developing other chronic diseases in the long term [53]. Although it has not yet been proven to be effective, antioxidants such as N-acetyl cystein could be beneficial in the early treatment of COVID-19.

## 5. Conclusions

In this study, DNA damage, some important oxidative stress parameters and cytokine levels significantly increased in SARS-CoV-2-positive patients when compared with healthy controls, and these parameters were increased more dramatically in severe patients. SARS-CoV-2 infection may increase oxidative stress and DNA damage and may alter immune responses, which might be important in the pathophysiology of the disease.

The short- and long-term effects of COVID-19 on human health are not clear yet; however, since coronaviruses can induce oxidative stress and DNA damage and can impair DNA repair mechanisms, there is the possibility of the risk of the later development of chronic diseases in recovered COVID-19 patients. Broad studies with larger study groups are needed to determine which components are the main factors in DNA damage and oxidative stress during SARS-CoV-2 infection. Increasing the sample size could result in more accurate data outputs. The understanding of these pathways could help to develop clinical treatments.

## Figures and Tables

**Table 1:** Characteristics of the study groups.

Characteristic	Patients with COVID-19 (*n* = 50)	Healthy Controls (*n* = 50)
Age (years) *	43.58 ± 16.17 (18–73)	41.16 ± 14.28 (18–75)
Body mass index *	25.23 ± 3.22 (19.53–31.59)	25.99 ± 3.62 (18.83–34.09)
Gender (*n* (%))		
Female	22 (44%)	22 (44%)
Male	28 (56%)	28 (56%)
Current cigarette smoker (*n* (%))		
Yes	17 (34%)	20 (40%)
No	33 (66%)	30 (60%)
Total pack-years of smoking *	8.23 ± 7.49 (0.20–25.0)	11.72 ± 19.84 (0.50–30.0)
Symptoms (*n* (%))		
Fever	20 (40%)	-
Cough	26 (52%)	-
Respiratory distress	12 (24%)	-
Hospitalization (*n* (%)) ^#^		
Yes (patients with severe symptoms)	12 (24%)	-
No (patients with mild symptoms)	38 (76%)	-

^#^ In the evaluation of hospitalization, CRP (>50 mg/L), d-dimer (>550 µg/mL), ferritin (>500 ng/mL), fibrinogen (>400 mg/dL) and lymphopenia (lymphocytes < 1 × 10^3^/µL) values were especially considered together with the symptoms. * The values are given as means ± standard deviation (min–max).

**Table 2:** Hemograms of the study groups.

Hemogram	Patients with COVID-19			Healthy Controls (*n* = 40)
	All Patients (*n* = 50)	Patients with Severe Symptoms (*n* = 12)	Patients with Mild Symptoms (*n* = 38)	
WBCs (10^3^/µL)	7.00 ± 2.14 (3.03–11.56)	6.87 ± 2.32 (3.22–10.8)	7.04 ± 2.08 (3.03–11.56)	5.72 ± 1.85 (3.2–9.8)
RBCs (10^6^/µL)	4.66 ± 0.81 (2.91–8.40)	4.59 ± 0.64 (3.33–5.60)	4.69 ± 0.85 (2.91–8.40)	4.33 ± 0.40 (3.54–5.21)
Hg (g/dL)	13.52 ± 1.66 (8.3–16.2)	12.89 ± 2.07 (9.40–16.2)	13.72 ± 1.46 (8.30–16.0)	13.89 ± 0.79 (12.5–15.7)
MCV (fL)	87.62 ± 4.96 (75.7–98.2)	86.05 ± 3.88 (78.4–92.4)	88.12 ± 5.15 (75.7–98.2)	89.35 ± 3.51 (82.6–95.2)
MCH (pg)	29.14 ± 2.24 (24.5–33.0)	28.02 ± 1.93 (24.5–31.4)	29.49 ± 2.22 (24.5–33.0)	30.83 ± 1.49 (28.0–34.0)
MCHC (g/dL)	33.23 ± 1.51 (28.6–35.8)	32.35 ± 1.49 (30.3–35.8)	33.51 ± 1.41 (28.6–35.6)	33.37 ± 1.42 (30.2–36.7)
HCT (%)	41.18 ± 4.59 (24.4–49.4)	40.51 ± 5.55 (29.1–49.4)	41.39 ± 4.23 (24.4–48.8)	40.86 ± 2.58 (36.5–47.3)
PLT (10^3^/µL)	261.2 ± 89.9 (99–526)	301.50 ± 116.60 (138–526) *	248.47 ± 75.20 (99–432)	220.86 ± 69.71 (112–372)
RDW–SD (fL)	41.02 ± 3.24 (35.2–57.0)	40.48 ± 2.22 (37.4–45.5)	41.20 ± 3.48 (35.2–57.2)	41.01 ± 2.19 (35.9–46.1)
PDW (fL)	15.93 ± 0.63 (14.3–17.6)	16.20 ± 0.46 (15.3–16.7)	15.85 ± 0.65 (14.3–17.6)	15.84 ± 0.86 (12.4–16.9)
MPV (fL)	9.70 ± 1.28 (6.11–14.4)	10.01 ± 1.14 (8.5–12.4)	9.60 ± 1.31 (6.11–14.4)	9.36 ± 1.01 (7.3–11.3)
PCT (10^3^/µL)	0.22 ± 0.08 (0.02–0.37)	0.22 ± 0.07 (0.10–0.31)	0.22 ± 0.08 (0.02–0.37)	0.26 ± 0.05 (0.13–0.35)
NEU (10^3^/µL)	3.77 ± 1.64 (0.1–8.52)	3.03 ± 1.09 (1.93–5.45)	4.01 ± 1.72 (0.10–8.52)	4.18 ± 1.04 (2.38–6.24)
MO (10^3^/µL)	0.44 ± 0.27 (0.02–1.10)	0.35 ± 0.26 (0.08–1.10)	0.47 ± 0.27 (0.02–1.01)	0.47 ± 0.28 (0.10–1.10)
LYM (10^3^/µL)	1.39 ± 0.78 (0.33–3.56) *	0.81 ± 0.34 (0.35–1.44) *^,#^	1.57 ± 0.79 (0.33–3.56)	2.65 ± 0.69 (1.01–4.10)
EOS (10^3^/µL)	0.09 ± 0.11 (0.0–0.43) *	0.02 ± 0.04 (0.0–0.16) *^,#^	0.11 ± 0.11 (0.0–0.43)	0.18 ± 0.10 (0.0–0.40)
BASO (10^3^/µL)	0.02 ± 0.01 (0.0–0.04)	0.02 ± 0.01 (0.0–0.03)	0.02 ± 0.01 (0.0–0.04)	0.03 ± 0.05 (0.01–0.30)

BASO, basophils; EOS, eosinophils; HCT, hematocrit; Hg, hemoglobulin; LYM, lymphocytes; MCH, mean corpuscular hemoglobin; MCHC, mean corpuscular hemoglobin concentration; MCV, mean corpuscular volume; MONO, monocytes; MPV, mean platelet volume; NEU, neutrophils; PCT, procalcitonin; PDW, platelet distribution width; PLT, platelets; RDW-SD, red-cell distribution width standard deviation; WBCs, white blood cells; RBCs, red blood cells. The values are given as means ± standard deviation (min–max). * *p* < 0.05, compared with healthy controls; # *p* < 0.05, patients with severe symptoms compared with patients with mild symptoms.

**Table 3:** Biochemical parameters of the study groups.

Parameter	Patients with COVID-19			Healthy Controls (*n* = 50)
	All Patients (*n* = 50)	Patients with Severe Symptoms (*n* = 12)	Patients with Mild Symptoms (*n* = 38)	
CRP (mg/L)	37.03 ± 58.44 (0.20–250) *	116.38 ± 71.41 (26.0–250) *^,#^	11.97 ± 16.34 (0.20–66.8) *	2.72 ± 2.01 (0.12–9.2)
Ferritin (ng/mL)	328.8 ± 396.2 (12.54–1893) *	738.1 ± 581.1 (67.2–1893) *^,#^	199.6 ± 174.0 (12.54–598.5) *	69.47 ± 48.83 (12.01–243.1)
d-Dimer (µg/mL)	578.7 ± 721.8 (85–4360) *	1356 ± 1117 (265–4360) *^,#^	333.1 ± 200.1 (85–983)	232.7 ± 109.8 (65–456)
Fibrinogen (mg/dL)	369.2 ± 169.1 (38–870) *	486.2 ± 204.2 (98–870) *	332.2 ± 137.0 (38–678)	260.6 ± 82.3 (78–412)
AST (U/dL)	27.64 ± 14.50 (13–84)	38.17 ± 20.79 (19–84) *	24.32 ± 9.70 (13–60)	21.84 ± 5.58 (10–36)
ALT (U/dL)	23.20 ± 12.26 (9–92)	23.17 ± 8.15 (13–39)	23.21 ± 13.30 (9–92)	18.40 ± 4.31 (11–30)

The values are given as means ± standard deviation (min–max). CRP, C-reactive protein; AST, aspartate transaminase; ALT, alanine aminotransferase. * *p* < 0.05, compared with healthy controls; # *p* < 0.05, patients with severe symptoms compared with patients with mild symptoms.

**Table 4:** Oxidative stress and immune parameters of the study groups.

Parameter	Patients with COVID-19			Healthy Controls (*n* = 50)
	All Patients (*n* = 50)	Patients with Severe Symptoms (*n* = 12)	Patients with Mild Symptoms (*n* = 38)	
MDA (nmol/L)	6.27 ± 7.76 (0.02–32.68) *	13.88 ± 9.87 (2.21–32.68) *^,#^	3.87 ± 4.94 (0.02–25.67) *	1.78 ± 1.05 (0.28–5.94)
GSH (ng/mL)	1.30 ± 1.33 (0.01–5.37)	1.55 ± 1.64 (0.01–5.09)	1.22 ± 1.20 (0.04–5.37)	1.29 ± 1.19 (0.01–5.36)
GPx (µU/mL)	48.66 ± 62.80 (0.27–277.56) *	103.00 ± 82.14 (5.71–277.56) *^,#^	31.50 ± 42.80 (0.27–217.20) *	10.75 ± 9.18 (0.24–38.15)
SOD (ng/mL)	10.44 ± 13.66 (0.01–54.43) *	22.11 ± 16.98 (3.31–54.43) *^,#^	6.76 ± 9.89 (0.01–50.88) *	2.60 ± 1.64 (0.01–7.62)
CAT (KU/L)	69.77 ± 75.14 (5.69–265.71) *	133.96 ± 84.53 (18.37–265.71) *^,#^	49.61 ± 58.98 (5.69–255.65) *	19.65 ± 10.22 (7.65–65.88)
IL-17 (ng/L)	52.29 ± 66.84 (4.08–316.09) *	113.96 ± 88.40 (16.01–316.09) *^,#^	32.82 ± 42.79 (4.08–241.36) *	14.81 ± 13.66 (4.57–99.62)
IL-23 (ng/L)	40.34 ± 53.78 (0.43–210.14) *	89.62 ± 62.97 (11.96–178.81) *^,#^	24.77 ± 39.29 (0.43–210.14) *	10.57 ± 4.69 (3.69–28.12)
IL-27 (ng/L)	136.8 ± 156.6 (20.37–526.22) *	292.73 ± 181.20 (32.63–512.52) *^,#^	87.52 ± 108.50 (20.37–526–22) *	35.19 ± 17.33 (10.04–90.93)

The values are given as means ± standard error mean (min–max). * *p* < 0.05, compared with healthy controls; # *p* < 0.05, patients with severe symptoms compared with patients with mild symptoms. MDA, malondialdehyde; GSH, glutathione; GPx glutathione peroxidase; SOD, superoxidase; CAT, catalase; IL-17, interleukin-17; IL-23, interleukin-23; IL-27, interleukin-27.

## Data Availability

The data presented in this study are available on request from the corresponding author. The data are not publicly available due to ethical restrictions.
